# Genome sequence-based species delimitation with confidence intervals and improved distance functions

**DOI:** 10.1186/1471-2105-14-60

**Published:** 2013-02-21

**Authors:** Jan P Meier-Kolthoff, Alexander F Auch, Hans-Peter Klenk, Markus Göker

**Affiliations:** 1 Leibniz Institute DSMZ – German Collection of Microorganisms and Cell Cultures, Braunschweig, Germany; 2 Eberhard-Karls-Universität, Tübingen, Germany

**Keywords:** *Archaea*, *Bacteria*, BLAST, DDH, GGD, GGDC, GBDP, Genomics, MUMmer, Phylogeny, Species concept, Taxonomy

## Abstract

**Background:**

For the last 25 years species delimitation in prokaryotes (*Archaea* and *Bacteria*) was to a large extent based on DNA-DNA hybridization (DDH), a tedious lab procedure designed in the early 1970s that served its purpose astonishingly well in the absence of deciphered genome sequences. With the rapid progress in genome sequencing time has come to directly use the now available and easy to generate genome sequences for delimitation of species. GBDP (Genome Blast Distance Phylogeny) infers genome-to-genome distances between pairs of entirely or partially sequenced genomes, a digital, highly reliable estimator for the relatedness of genomes. Its application as an in-silico replacement for DDH was recently introduced. The main challenge in the implementation of such an application is to produce digital DDH values that must mimic the wet-lab DDH values as close as possible to ensure consistency in the Prokaryotic species concept.

**Results:**

Correlation and regression analyses were used to determine the best-performing methods and the most influential parameters. GBDP was further enriched with a set of new features such as confidence intervals for intergenomic distances obtained via resampling or via the statistical models for DDH prediction and an additional family of distance functions. As in previous analyses, GBDP obtained the highest agreement with wet-lab DDH among all tested methods, but improved models led to a further increase in the accuracy of DDH prediction. Confidence intervals yielded stable results when inferred from the statistical models, whereas those obtained via resampling showed marked differences between the underlying distance functions.

**Conclusions:**

Despite the high accuracy of GBDP-based DDH prediction, inferences from limited empirical data are always associated with a certain degree of uncertainty. It is thus crucial to enrich in-silico DDH replacements with confidence-interval estimation, enabling the user to statistically evaluate the outcomes. Such methodological advancements, easily accessible through the web service at http://ggdc.dsmz.de, are crucial steps towards a consistent and truly genome sequence-based classification of microorganisms.

## Background

DNA-DNA hybridization (DDH) is a wet-lab method currently still used as the taxonomic gold standard for species delineation in *Archaea* and *Bacteria*. If the genomic DNA of two respective organisms reveals a DDH similarity of below 70% this is the main argument to regard them as distinct species and vice versa [1,2]. DDH is widely considered as tedious, laborious and potentially rather error-prone [3,4]. Moreover, in contrast to genome sequencing it does not return more information than the DDH value itself and, as a consequence, it is impossible to work incrementally by re-using data.

The DDH technique is currently established in only a few specialized labs (mainly microbial service collections) and, because it is prone to experimental deviation, requires several experimental repetitions to determine the statistical confidence of that experiment. For instance, regarding species delimitation in microbiology, the relevant question is whether or not the DDH value is significantly below or above 70%. This is particularly important in the context of a polyphasic approach, in which the evidence from DDH has to be traded off against other criteria such as phenotypic measurements [5]. DDH experiments can be omitted in descriptions of novel species only if the 16S rRNA sequence similarity is below a certain threshold, indicating that DDH values above 70% cannot be expected [2].

The increasing availability of genome sequences thus triggered the development of computational techniques to replace wet-lab DDH [6-8]. These were expected to provide the deepest possible resolution for differentiation, to ensure much higher reproducibility of the results and to allow incremental work by filling databases with type-strain genome sequences [4]. But unless high correlations with wet-lab DDH, and precise models for estimating DDH or at least DDH-analogous species boundaries from genome-to-genome comparisons, were available, the newly calculated values were not comparable to the previous ones and could yield largely deviating species-boundary estimates and, thus, an inconsistent microbial taxonomic classification. Hence, for obvious reasons the literature on in-silico replacements for DDH considered correspondence with wet-lab DDH values as optimality criterion. As a consequence, regression and/or correlation analyses with wet-lab DDH values were used throughout for the calibration and optimization of the in-silico replacement methods [6-8].

In view of the technical problems and progress the relation between the wet-lab DDH procedure and digital estimation of DDH equivalents reminds very much to what happened some 30 years ago when DNA:rRNA cross-hybridization melting curves [9,10] were replaced by 16S rRNA sequences, which supported a significant progress in microbial phylogeny [11].

The Genome Blast Distance Phylogeny approach (GBDP) was originally devised as an approach for the inference of phylogenetic trees or networks from a given set of wholly (or even incompletely) sequenced genomes [12], and was subsequently revisited and enhanced [8,13-16]. The underlying principle is as follows: in the first step two genomes A and B are locally aligned using tools such as BLAST[17], which produce a set of high-scoring segment pairs (HSPs; these are intergenomic matches). In the second step, information contained in these HSPs (e.g., the total number of identical base pairs) is transformed into a single genome-to-genome distance value by the use of a specific distance formula. Phylogenetic trees can then be inferred from such distance matrices using standard techniques such as neighbour joining [18]. These methods are robust even in the presence of a significant amount of paralogous genes, large repeats and reduced genomes [12], as well as low complexity-regions within the sequences [16]. GBDP could also be applied to proteomic data [13] and even to single genes [19].

A further use of GBDP was recently evaluated, namely to infer digital equivalents for DDH values [8,16]. These turned out to successfully mimic the wet-lab hybridization results, providing higher correlations with an empirical set of DDH values than antecedent genome sequence-based methods [6] and being able to deal with rather incomplete genomes [8]. Microbiologists can make use of GBDP by means of a free web service at http://ggdc.dsmz.de[16] for submitting genome pairs and receiving DDH analogues as well as model-based DDH estimates. Such values on the original scale of wet-lab DDH measurements have the practical advantage that the well-known 70% threshold can still be applied [8], even though they are mathematically equivalent to in-silico DDH analogues that use a scale of their own, and accordingly represent a novel species-delimitation threshold [6,7].

The first goal of the present study is to improve DDH estimation from genome-sequence comparisons by using a more comprehensive empirical database and by considering a broader range of numerical data transformations and statistical models. Previous studies were limited to regression models of the untransformed data and thus presupposed a linear relationship between wet-lab DDH and the results of genome-sequence comparisons [6-8]. But this assumption might be unjustified, and the inspection of more complex models ([20], pp. 58–79) and distinct data transformations [21,22] has frequently been recommended. In addition to suboptimal fits, linear models can lead to DDH predictions below 0% and above 100% similarity if the underlying (dis-)similarity values are close to the upper bound (1 if distances are not logarithmically transformed) or the lower bound (0), respectively.

The second goal of these examinations is to obtain confidence intervals for in-silico DDH values – an indicator showing taxonomists how uncertain a reported value is, especially if it is close to the 70% boundary. Even though it is safe to assume a priori that digital DDH values display much less variability than wet-lab DDH experiments given the high sequence coverage that can be obtained with state-of-the-art sequencing technology [4], it is of interest whether, and how, confidence intervals can be calculated for the in-silico replacement methods, too. Hence, GBDP was further extended by integrating resampling techniques for calculating a confidence interval per point estimate (i.e., pairwise distance). Bootstrapping [23] and jackknifing [24] are well-known and robust resampling techniques for estimating the variance of a sample. But uncertainty might additionally or mainly be caused by the empirical modeling of the relationship between DDH and genome-sequence comparisons (see below), and the relative proportions of resampling- and model-based confidence intervals need to be assessed. Confidence intervals would render GBDP the first in-silico procedure to infer DDH analogs that can be statistically evaluated, which is particularly important in the context of the polyphasic approach to microbial taxonomy (see above).

The third topic of this study is to broaden the range of considered GBDP distance functions. Whereas [8] already investigated a much more diverse range of in-silico DDH analogues than previous publications on DDH-replacement methods [6,7], here GBDP is further enriched with so-called “coverage distances” [12] (and bootstrapping and jackknifing for this algorithm). The performance of the novel GBDP implementation could thus be assessed under 4350 distinct settings (ranging from the local alignment tools and their settings to the distance functions), requiring a total of 136 million individual genome comparisons (including bootstrapping and jackknifing), and the overall best-performing settings determined. The effects of the parameters used for the calculation of intergenomic distances on the resulting correspondence with the DDH values was also investigated in detail using multiple regression.

The results of this study are thus likely to contribute toward progress in using the comprehensive information encoded in entire genomes for the taxonomy of prokaryotes.

## Methods

### Extended benchmark data set

The DDH benchmark data set was extended compared to previous studies aiming at an increased precision and significance of the ranking of the genome-to-genome distance methods and the models for the conversion to DDH values. In detail, the here used data set (henceforth called “DS1”) comprised 156 unique genome pairs along with their respective DDH values: 62 from Goris et al. [6], 31 from the GOLD database [25], and 63 from Richter et al. [7]. Only the first two sources had been considered in a previous publication on GBDP as DDH replacement [8].

If several DDH/ANIb/ANIm/Tetra values were present for a single genome pair, they were averaged. A single genome pair showed a DDH value above 100% similarity (i.e., 100.9% between *Escherichia coli* O157:H7 EDL933 and *Escherichia coli* O157:H7 Sakai). As it biologically made not much sense this value was set to 100% to maintain proper input data for some of the statistical models (see below). Another genome pair (*Thermotoga maritima* MSB8 and *Thermotoga petrophila* RKU-1) had a contradicting relation between its DDH value (16.9%) and the genome based distance/similarity measures (GBDP, ANI, ANIb, ANIm and Tetra) on the other hand [7]. Following [7], this questionable data point was excluded from the correlation analyses. The full list of genome pairs used in this study is found in the Additional file 1.

To detect significant deviations, if any, between the new and the previous GBDP implementation, the data subset “DS2” was created, containing only the previously available data points [8]. For comparing GBDP with the first ANI implementation, data subset “DS3” comprised the 62 data points in common between [6,8]; for comparison with the JSpecies study, subset “DS4” contained only the 98 DDH values in common between [7,8].

### The GBDP principle, and its technical update

To motivate the upcoming changes such as the addition of support for BLAST+[26] and the completion of the implementation of the so-called “coverage” algorithm [12], the major steps within the GBDP pipeline [8,12,13] are summarized in the following.

The pipeline is primarily subdivided into two phases. First, a genome X is BLASTed against a genome Y and vice versa (here, the term “BLASTed” denotes the application of one out of six supported local-alignment programs; a full list of these programs is found in Additional file 2). BLAST+ has recently been added to the list of available programs, because it provides substantial speed improvements for long queries and database sequences [26]. The alignment process is done in one pass using all the available sequence information of both respective genomes, i.e., GBDP does not require the sequences to be artificially cut into pieces as do other approaches [6].

The resulting matches between both genomes are called high-scoring segment pairs (HSPs) and represent local alignments that are considered statistically significant if the associated expect value (e-value) is sufficiently low [27] (reliable thresholds are usually equal or less than 10^−2^, but GBDP conducts the filtering itself, and its effect is explicitly addressed below).

In the second phase, these matches are transformed to a single distance value *d*(*X*,*Y*) by applying one out of ten available distance formulae *d*_0 _to *d*_9_. To describe these, the following definitions are required:

(1) $$\begin{array}{lll} \text{XY} & \;:= & \text{BLAST}\text{run using genomes X (subject)}\;\\ & & \;\text{and Y (query)}\; \end{array}$$

(2) $$\begin{array}{lll} I_{\text{XY}} & \;:= & \text{sum of identical base pairs over all HSPs} \end{array}$$

(3) $$\begin{array}{ll} \;H_{\text{XY}}:= & \;\text{total length of all HSPs} \end{array}$$

(4) $$\begin{array}{ll} \;\lambda (X,Y):= & \text{sum of both genomes’ lengths}\; \end{array}$$

(5) $$\begin{array}{ll} \;\lambda_{\text{min}}(X,Y):= & \text{twice the length of the smallest genome}\; \end{array}$$

The web service at http://ggdc.dsmz.de makes use of these formulae; the other GBDP formulae are minor variants [8,12,13] and are found in Additional file 3:

(6) $$\begin{array}{ll} d_{0}(X,Y) & =1-\frac{H_{\text{XY}}+H_{\text{YX}}}{\lambda (X,Y)}\; \end{array}$$

(7) $$\begin{array}{ll} d_{4}(X,Y) & =1-\frac{2\cdot I_{\text{XY}}}{H_{\text{XY}}+H_{\text{YX}}}\; \end{array}$$

(8) $$\begin{array}{ll} d_{6}(X,Y) & =1-\frac{2\cdot I_{\text{XY}}}{\lambda (X,Y)}\; \end{array}$$

Each was devised to consider distinct aspects of intergenomic relationships. Formula *d*_6 _preserves most information, because it is some kind of combination of *d*_0 _and *d*_4_. It also performs best in a phylogenetic context [13]. However, *d*_4 _is immune against problems caused by incompletely sequenced genomes, as it does not consider the genome lengths [8]. It follows from *d*_0 _to *d*_6 _that similarity instead of dissimilarity (distance) values could be easily obtained by subtracting the distances from 1 ([28], pp. 252–259) [8,13]. This would be mathematically analogous for the subsequent correlation analyses because only the sign changed, but using distances is more convenient for inferring GBDP trees [12,13].

**Figure 1:**
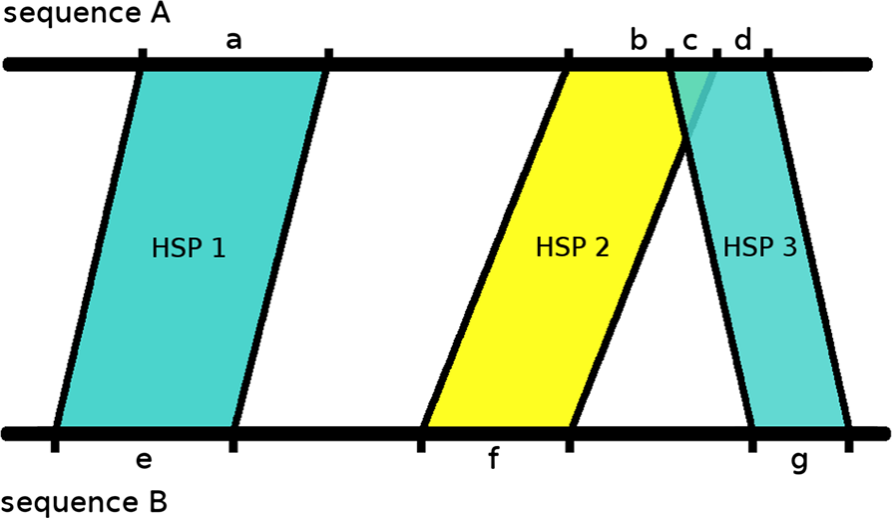
**An example of a hypothetical HSP layout between two genomes A and B as produced during the GBDP alignment phase.** Subsequences that are part of an HSP in either A or B are labeled with small letters a-g. A special case is represented by segment “c” where both HSP 2 and HSP 3 are overlapping. GBDP’s algorithms are programmed to handle these distinctly, i.e., (i) by simply completely omitting the smaller HSP 3 (“greedy” algorithm), (ii) by omitting only segment “c”, i.e. trimming HSP 3 (“greedy-with-trimming” algorithm), or (iii) by merging information from both HSPs regarding the overlapping segment (“coverage” algorithm). (Figure redrawn from [15]).

However, in practice, at least some HSPs from a BLAST run between X and Y are very likely to overlap (see Figure 1, segment “c”), mainly because paralogous genes can be present [12]. With respect to definitions 2 and 3 these overlapping segments would introduce a bias in the resulting distance value, because they would be considered more than once in the calculations. For this reason, GBDP includes three distinct approaches for filtering HSPs and, thereby, resolving these conflicts before one of the aforementioned 10 distance formulae is applied: “greedy”, “greedy-with-trimming” and “coverage”. For technical reasons, “coverage” was previously [8] only available in connection with distance formulae *d*_0_- *d*_3_, but the missing ones were implemented in the course of this study. **Greedy** If two or more HSPs are overlapping in a specific segment, this algorithm omits all HSPs except the largest one (e.g., HSP 3 in Figure 1). Some information is thus lost, but this approach is computationally faster than the next one.**Greedy-with-trimming** Here, only the overlapping parts of HSPs in either genome are removed, as, for instance, segment “c” in Figure 1. This preserves more information and proved to be valuable in phylogenetic inference from genomes with large numbers of repeats [12].**Coverage** The content of the HSPs is mapped to two vectors (henceforth called coverage vectors) representing the positions within either genome. Concerning definition 3 this was implemented by considering a position within a genome as covered by an HSP if there is at least one HSP that covers it [12], whereas definition 2 in conjunction with coverage works by assigning the highest identity among all HSPs that overlap in a certain genome segment to the positions within this segment. In detail, a coverage vector, as a vector of length |*G*|, is (formally) computed for each genome as follows: Let *h* be a matching segment of an HSP and *I* its proportion of identical characters. Then segment *h* beginning at position *i* and with length *l* can be displayed as a vector of length |*G*| (the genome length) as follows:

(9) $$h=(0_{1},\ldots ,0_{i-1},I_{i},I_{i+1},\ldots ,I_{i+l-1},0_{i+l},\ldots ,0_{\left|G\right|})$$

Each vector position which is not covered by *h* is set to 0, while each covered position is set to *I*. Further, let PMAX (“parallel maxima”) be a function that reduces a list of equal-length vectors to a single vector by determining the maximum for each vector position:

(10) $$\;\begin{array}{cc} \text{PMAX}(a,b,\ldots ) & :=(\text{max}(a_{1},b_{1},\ldots ),\ldots ,\text{max}(a_{n},b_{n},\ldots ))\\ \text{for}\;a & \;=(a_{1},\ldots ,a_{n})\;\text{and}\;b=(b_{1},\ldots ,b_{n}) \end{array}$$

In the case of the coverage vectors n equals the length of the respective genome (i.e., |*G*|). Then, the coverage vector *v*_*G*_ = PMAX(*h*_1_,…,*h*_*k*_) is calculated by applying PMAX to the list of *k* HSP segments within genome *G*. Analogously, a second vector is calculated for the HSP intervals within the second genome. The numerator of the identity- based dissimilarities (see formulae 7 and 8 above) can then be calculated by using the sums over all vector positions:

(11) $$I_{\text{XY}}=\frac{\frac{\sum (v_{X})}{\left|v_{X}\right|}+\frac{\sum (v_{Y})}{\left|v_{Y}\right|}}{2}$$

Analogously, the number of genome positions covered by HSPs, *H*_*XY *_and *H*_*YX*_, as used in formulae 6 and 8, is calculated by counting all non-zero positions in the vectors *v*_*X *_and *v*_*Y*_, respectively. The coverage method is faster than the other two approaches, because there is no overhead caused by HSP sorting and/or trimming algorithms.

### Conducting genome comparisons for the correlation analysis

A correlation analysis was conducted to show the overall performance of the GBDP method and to yield the best GBDP parameter setup. Six local-alignment tools were tested for genome comparisons: BLAST+[26], NCBI-BLAST[17], MUMmer[29], BLAT[30], WU-BLAST[17] and BLASTZ[31]. These were not only used to conduct six genome comparisons per available genome pair but were also applied in several passes, each time changing a chosen parameter, presumably affecting the local alignment and thus potentially improving the correlation with DDH. A special focus was on finding influential parameters for BLAST+ because it is one of the technically most advanced local-alignment tools available [26]. Moreover, all available distance functions and HSP filtering approaches were applied to each genome comparison (see above). Thus, all in all 4350 distinct settings were investigated, i.e., the product of 145 alignment settings, three algorithms for dealing with overlapping HSPs, and ten distance formulae. This resulted in a total of 136 million GBDP-based genome comparisons which had to be conducted. Additional file 2 provides a complete overview on these settings and numbers.

In general, studies of that kind are computationally challenging, because a huge number of input and result files need to be processed. This gave rise for equipping the method with an extension allowing it to be executed on compute clusters [32-34].

### Analyzing correlations between intergenomic distances and DDH values

According to [8] Pearson’s *ρ* ([35], pp. 533) and Kendall’s *τ* ([28], pp. 198–199) were computed for all distinct GBDP settings and the DDH values, respectively. The necessary analysis pipeline was implemented as an R[36] script and applied to the previously described main data set DS1 and its subsets DS2-4. As GBDP-derived values are distance measures, whereas ANI values are similarities between genomes, the correlation coefficients’ sign had to be inverted accordingly to allow for the direct comparison of the performance of GBDP, ANI and JSpecies. The full list of the genome pairs and their associated DDH and ANI values is found in Additional file 1.

For GBDP, the influence of four predictor variables was tracked by means of multiple linear regression ([37], pp. 387-448): alignment method, algorithm for treating overlapping HSPs, distance formula and e-value filter method (Additional file 2). The identification of the least complex model that still explained most of the variation in the data was conducted with the R package MASS[38] under both forward variable selection and backward elimination. For an interpretation of possible interaction effects, the effects package [39] for R was used. Additionally, each predictor variable’s relative importance index [40] was computed.

### GBDP bootstrapping and jackknifing

To obtain confidence-interval (CI) estimates, GBDP was augmented with sampling with replacement (bootstrapping [23]) and sampling without replacement (jackknifing [24]), by default using 50% deletion in each replicate. For the variants of GBDP that use a filtering approach for removing overlapping (parts of) HSPs, namely the “greedy” and “greedy-with-trimming” algorithms (see above), the implementation treats individual HSPs as the observations for resampling. After applying the filtering step, the original distance (the point estimate) is inferred from the set of filtered HSPs using one of the distance formulae described above. Then the set of HSPs is bootstrapped or jackknifed, and in each replicate a distance value is calculated using the same formula. For the “coverage” algorithm (see above), resampling was implemented by bootstrapping (or jackknifing) the constructed coverage vectors. For the evaluation of the methods, 100 replicates were used throughout.

The dependency of the resulting bootstrapping and jackknifing CIs on each genome pair’s original distance (point estimate) was investigated, as well as the effect of the GBDP method on the replicates’ variation. The latter was assessed via the median of the variation coefficients [41] calculated for each genome pair and its respective replicates. It was also evaluated to what extent the CI width was affected by the distribution of both numbers and sizes of HSPs derived from a respective pair of genomes. This was done for a selected, well performing BLAST+ method (see below). To assess the amount of uncertainty indicated by the bootstrap/jackknife CIs in DDH prediction, the bounds of each CI were transformed to DDH (in addition to the distance point estimate) using the investigated prediction model. Thus, the relationship between (i) the model’s CI estimating the uncertainty of DDH prediction, as detailed in the next section, and, (ii) the bootstrap/jackknife CI translated to DDH scale was assessed for each observation.

### DDH prediction using sophisticated statistical models

The problems caused by linear models (see above) for predicting DDH via intergenomic distances can be solved by more sophisticated statistical models such as generalized linear models (GLMs) [42], generalized additive models (GAMs) [43] and Loess models [44] and identifying the one that provides the most robust predictions ([20], pp. 58–79). All models used DDH values as response variable and the corresponding intergenomic distance values as predictor variable. Among the novel models assessed in this study, GLMs came to our attention for several reasons: (i) they do not require the response variable to be normally distributed, (ii) the response predictions (here: DDH) would be bound within a fixed interval between a 0 and 100% and (iii) the predictor variable does not need to have a constant variance. The latter was especially important because, in our case, as usual for proportion data, the distance values are strictly defined in a range between 0 and 1, thus leading to a decrease in variance when approaching these boundaries, causing a dependency of the variance on the mean ([37], pp. 571).

GLMs belong to the parametric modeling techniques and make assumptions about the underlying distribution. For proportional response data as present here a binomial distribution is recommended ([37], pp. 515). One benefit of such a logistic regression is the variance-stabilizing effect on the response variable (removal of heteroscedasticity). DDH response data was appropriately converted to represent the number of failures and successes of an event ([37], pp. 574). Another special type of GLM was constructed by changing the response from DDH proportions to a binary response variable ([37], pp. 593–610). For any given intergenomic distance such a model yields the probability of whether or not it corresponds to a DDH value ≥ 70%. Finally, a non-parametric Loess smoother was also evaluated, as well as generalized additive models [45] but neither yielded better results than the GLMs (data not shown).

To assess whether the fit of the overall model (determined by the model’s residual deviance) could be further improved, a log transformation was applied to the explanatory variable [21,22], and/or a variance-stabilizing arcsine transformation was applied to the response variable (see [37], pp. 570 and [35], pp. 386). In standard linear-regression models the coefficient of determination (*R*^2^) provides a measure of how well future outcomes are likely to be predicted by a certain linear model. As GLMs do not provide *R*^2^ for model diagnosis, we checked for potential overdispersion (i.e., extra, unexplained variation) ([37], pp. 522) and where applicable used the Akaike information criterion [46] to measure the relative goodness of the GLM fits. Graphical evaluation of the model fits was done using the R package ggplot[47].

The performance of the model types and data transformations was also assessed by computing error ratios in DDH prediction. For each of the 4350 GBDP settings we calculated the models (under DS1) and compared the DDH predictions with the respective wet-lab DDH value. In a second pass, we calculated the model’s error ratio by assessing the number of false positives (i.e., a predicted DDH value equal or above 70% corresponding to a real DDH value below that threshold) and false negatives (i.e., a predicted DDH value below 70% corresponding to a real DDH value equal or above that threshold) relative to the total number of observations. To investigate the impact of the extension of the empirical data set, we chose the best-performing GBDP method from this study and fitted the aforementioned models to both the full data set (DS1) and the reduced one (DS2).

## Results

### Performance of methods and settings in mimicking wet-lab DDH

**Figure 2:**
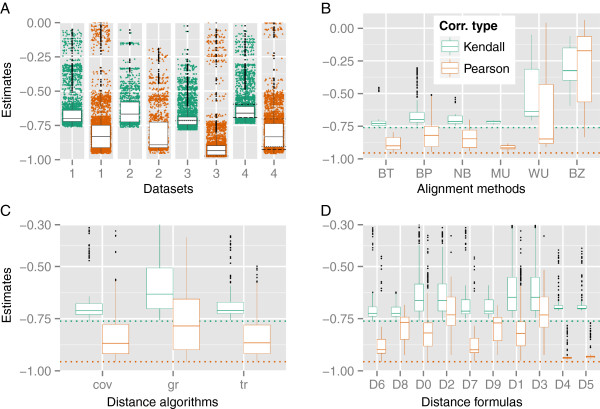
**Results of the correlation analyses between GBDP-derived distances and DDH as opposed to the correlations between ANI and DDH.A:** The performance of both GBDP and ANI regarding their correlation with wet-lab DDH is shown. The boxplots visualize the correlation results for the data sets DS1-4, created for conducting fair comparisons between GBDP, the original ANI implementation [6] and JSpecies [7 ](green circles: Kendall’s *τ*; orange triangles: Pearson’s *ρ*). For the purpose of an easier visualization, the scale has been bound by 0 and -1, thus omitting a few outliers greater than 0, and the sign of correlation values involving similarities was inverted. The correlation coefficients between ANI and DDH are highlighted by horizontal lines, either dotted (DS3, ANI; DS4, ANIm), dot-dashed (DS4, ANIb) or long-dashed (DS4, Tetra). **B: **GBDP correlations (DS1) dependent on the alignment tools used: BLAT (BT), BLAST+ (BP), NCBI-BLAST (NB), WU-BLAST (WU), MUMmer (MU) and BLASTZ (BZ). The dotted lines represent the globally best correlation (i.e., the most negative one), and the boxplots are sorted increasingly by their most negative Kendall coefficient, i.e., the best setting can be found at the leftmost position. The same applies to C and D. **C:** Results for DS1 dependent on the algorithms “coverage” (COV), “greedy” (GR) and “greedy-with-trimming” (TR). **D:** Correlations based on DS1 dependent on distance formulae *d*_0_ - *d*_9_. For obvious reasons, the distance formulae *d*_0_, *d*_1_, *d*_4_, *d*_6_ and *d*_7_ yielded the same Kendall correlations as their logarithmized variants *d*_2_, *d*_3_, *d*_5_, *d*_8_ and *d*_9_.

Figure 2A depicts the overall correlation results obtained with the data sets DS1-DS4. Figure 2B-D contain details of the contribution to the overall correlations of (i) local-alignment methods, (ii) algorithms and (iii) distance formulae. The correlation coefficients for all tested GBDP methods and data sets are included in Additional file 4. A preselection of well-performing GBDP settings are presented in Table 1 along with correlation values for the dis- tinct ANI implementations. Regarding BLAST+, the best Kendall’s *τ* (-0.752) resulted from an alteration of the word length from 11 (default for nucleotide data) to 46, the use of the “coverage” algorithm and either *d*_6 _or *d*_8 _as distance formulae (no e-value filtering yielded the same results as e-value thresholds of 10^−1^, 10^−2 ^and 10^−8^). The same setting except *d*_4 _(which is preferable when dealing with incomplete genomes [8]) instead of *d*_6 _or *d*_8 _yielded a *τ* of -0.677 and a Pearson’s *ρ* of -0.935. These results confirmed and slightly improved on the results from a previous study of the (smaller) data set DS2 [8], which also yielded BLAT and distance formulae *d*_6 _and *d*_8 _as the combination with the globally best Kendall and Pearson correlation (-0.763 and -0.954, respectively), the only exception being that “greedy-with-trimming” instead of “coverage” (which was not fully implemented at that time) performed best.Regarding the correlation results, the most successful GBDP methods outperformed ANIm, ANIb and Tetra to an even higher degree than ANI (Figure 2A and Table 1).

**Table 1:** Preselection of well-performing GBDP methods from the correlation analysis

	**Correlations**		**Settings**			
**Dataset**	**Type**	**Estimate**	**Alignment tool or method**	**E-value filter**	**Algorithm**	**Formula**
DS1	Kendall	-0.761	BLAT	10	Coverage	*d*_6_, *d*_8_
		-0.752	BLAST+ (WL46)	10	Coverage	*d*_6_, *d*_8_
		-0.677	BLAST+ (WL46)	10	Coverage	*d*_4_
	Pearson	-0.956	BLAT	10	Greedy	*d*_6_
		-0.956	BLAT	10^−2^	Trimming	*d*_6_
		-0.946	BLAST+ (WL38)	10	Coverage	*d*_4_
		-0.935	BLAST+ (WL46)	10	Coverage	*d*_6_, *d*_8_
DS2	Kendall	-0.763	BLAT	10	Coverage	*d*_6_, *d*_8_
	Pearson	-0.954	BLAT	10	Coverage	*d*_6_, *d*_8_
DS3	Kendall	-0.783	BLAST+ (WL38)	any	Coverage	*d*_6_, *d*_8_
		-0.717	ANI	-	-	-
	Pearson	-0.980	MUMmer (MR20)	-	Greedy	*d*_0_, *d*_6_
		-0.973	ANI	-	-	-
DS4	Kendall	-0.737	BLAT	10, 10^−2^	Coverage	*d*_6_, *d*_8_
		-0.735	BLAST+ (WL45)	any	Coverage	*d*_6_, *d*_8_
		-0.693	Tetra	-	-	-
		-0.598	ANIb	-	-	-
		-0.594	ANIm	-	-	-
	Pearson	-0.957	BLAT	10^−2^	Greedy	*d*_6_
		-0.904	ANIm	-	-	-
		-0.703	ANIb	-	-	-
		-0.693	Tetra	-	-	-

Juxtaposition of DDH correlation values for best-performing GBDP methods as well as (i) ANI [6] and (ii) JSpecies[7] implementation (ANIm, ANIb, Tetra). The content of the respective data sets DS1-DS4 is described in Materials and Methods, whereas the full table with all correlation results is found in Additional file 4. For convenience, the correlation coefficients’ sign of the ANI values is inverted to allow for the direct comparison toward GBDP (-1 is the optimal value). Abbreviations used: WL (wordlength) and MR (mumreference). The listed BLAT runs were all conducted under the same settings (minScore=30, minIdentity=90, tileSize=12). GBDP-based correlations surpass any of the ANI implementations throughout the respective data sets.

The most influential GBDP parameters were assessed via a multiple regression under two types of model selection (forward selection and backward variable elimination) with both resulting in the same full regression model. The latter contained the independent variables “alignment tool”, “distance algorithm” and “distance formula” and all possible interaction terms between them (Additional file 5). That is, only “e-value filter method” got eliminated as it could not explain a significant amount of variation in the data. This was confirmed by the relative importance index (see Additional file 6, pictures 1 and 2) and an ANOVA (here, p-values were 0.8136 for Pearson’s *ρ* as response variable and 0.6594 for Kendall’s *τ*). Additional file 6 (pictures 3-6) shows the interaction effects and the impact of all independent variables.

### Confidence intervals via bootstrapping or jackknifing

**Figure 3:**
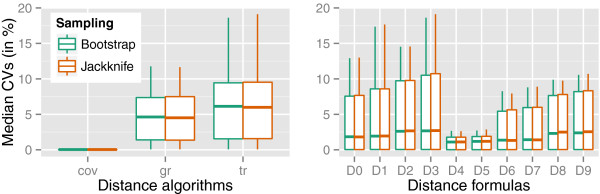
**Distributions of the median coefficients of variation of intergenomic distances obtained by resampling GBDP.** The depicted distributions were determined by grouping the median coefficient of variation (CV) for each setting by either algorithms (**left**; “greedy”, gr; “greedy-with-trimming”, tr; “coverage”, cov) or formulae (**right**).

The effect of the GBDP settings on the bootstrapping and jackknifing confidence intervals as assessed by the aggregated coefficients of variation (CVs) is shown in Figure 3. The “coverage” algorithm resulted in low CVs with little variation (Figure 3A), while the variation induced by “greedy-with-trimming” was the most pronounced one, and the CVs were on average much higher, but only slightly higher than for the “greedy” variant (the median CV of all GBDP settings was around 6%; data not shown). The effect of the distance formulae on the CVs is shown in Figure 3B. The median CV was around 1% for formulae *d*_4_ - *d*_7 _and around 2.5% for the remaining ones. Bootstrapping and jackknifing had nearly the same effect on the CV’s distribution (compare Additional file 6, pictures 7 and 8).

**Figure 4:**
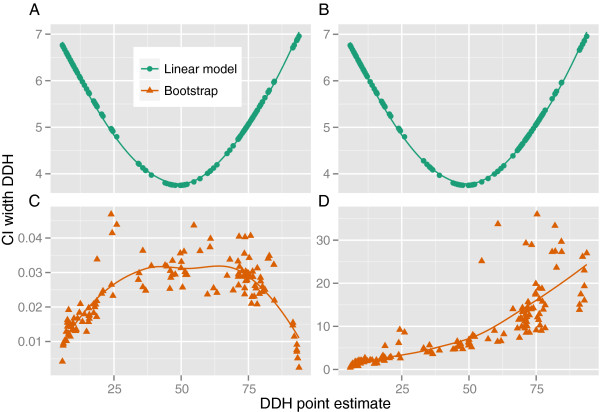
**Juxtaposition of confidence-interval widths for both model based DDH predictions and those induced by bootstrap replicates.** Distances were calculated under the selected well-performing GBDP method (see main text) either using the “Coverage” algorithm (**A** and **C**) or “Greedy-with-Trimming” (**B** and **D**). For each distance value the respective DDH predictions were made with a simple linear regression model (x-axis) and the widths of their 95% CIs determined accordingly (y-axis).

Figure 4 shows the relationship between CIs of the in-silico DDH values calculated by either bootstrapping or via the model from the intergenomic distances, both obtained with the chosen GBDP settings from data set DS1 (but either the “coverage” or the “trimming” algorithm). A simple linear regression model based on data set DS1 was used (response variable: DDH, explanatory variable: intergenomic distances). Under “coverage”, the length of the CI decreased when the distances approached either their lower or upper bound; the CI widths under “trimming” were two orders of magnitude higher and continuously increased toward the lower bound. Both plots of the model-based CIs in dependency of the point estimates had an U-like shape; the size of the model-induced CIs was an order of magnitude higher than the one of the “coverage” bootstrapping CIs and an order of magnitude smaller than the size of the “trimming” bootstrapping CIs. For the same settings, the dependency of the confidence-intervals’ width (CIs) directly on the sizes of the distance point estimates is shown in Additional file 6 (picture 9), confirming the results depicted in Figure 4C and 4D. A fit by a Loess smoother revealed only minor differences between bootstrapping and jackknifing.

The relationship between the intergenomic distance and the underlying set of HSPs obtained by comparing the respective pair of genomes is presented in Additional file 6 (picture 10). In brief, the lengths of the HSPs are more unevenly distributed for more similar genomes (smaller distance values).

### Models for DDH prediction and species delineation

**Figure 5:**
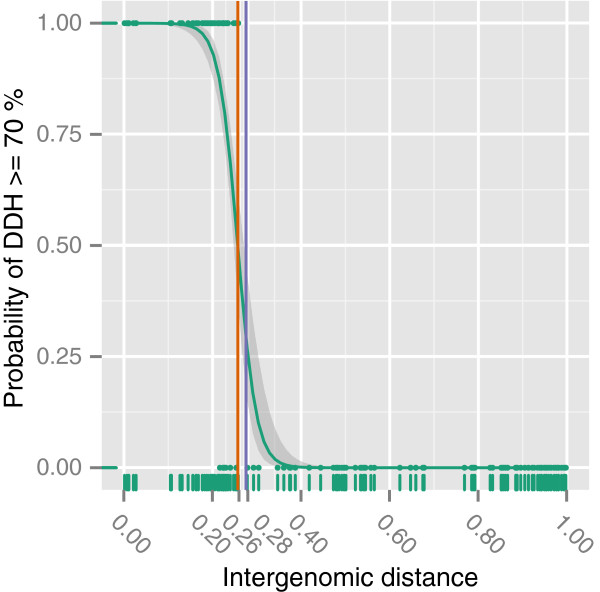
**GLM with a binary response variable.** The curve depicts the predictions from the model for the selected well-performing GBDP settings (see main text). The y-axis indicates the GBDP-derived probability that a DDH value is above 70%, indicating that two genomes represent organisms of the same species. The orange vertical line marks the distance threshold for species delineation as provided by the GLM, i.e., denoting a probability of 0.5. The blue vertical line marks an alternative error ratio-based distance threshold as presented in our previous article [8].

Figure 5 shows the results of the generalized linear model (GLM) with a binary DDH response variable that indicates whether an intergenomic distance would yield a wet-lab DDH value above 70%. The distance threshold yielding a probability of 0.5 was marked as this denotes a reasonable model-based threshold for species delineation. The model showed a steep transition area in which the probability rapidly dropped with an increase in intergenomic distances. Under a well-correlating {BLAST+} setting (i.e., second one in Table 1 with formula *d*_6_), it yielded a distance threshold of 0.258 below of which all respective genome pairs could safely be considered to be the same species (because their DDH value would be above 70%). An alternative way of computing such a distance threshold was introduced in [8] and is based on choosing the distance value associated with the lowest error ratio; if applied to the same data this method resulted in a threshold of 0.276 (Figure 5). The following figures, however, show that the newly calculated cutoff fits better to the other models applied.

**Figure 6:**
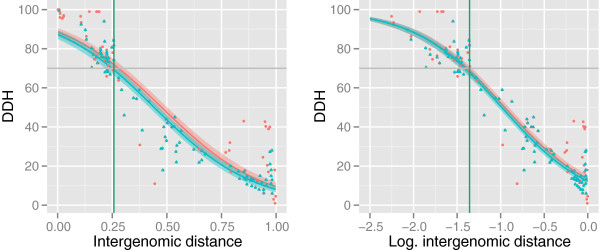
**Comparison of generalized linear models and data transformations for DDH prediction.** All model fits were based on distances calculated with the selected well-performing GBDP method (see main text). The models were either inferred from (i) the complete data set DS1 (red and blue curves, red circles and blue triangles) or (ii) the reduced data set [8] DS2 (blue curve and blue triangles). The green vertical line indicates the 50% probability threshold as calculated by the GLM for binary response data (see Figure 5). **Left:** GLMs (generalized linear models with quasi-binomial error family) based on DDH proportion data as response and untransformed distance values as predictor variable. **Right:** GLMs (generalized linear models with quasi-binomial error family) based on DDH as response and logarithmized distance values as predictor variable.

The results for the GLMs using wet-lab DDH values as response variable are shown in Figure 6; Additional file 6 (picture 11) contains the results for the linear models. Each part of these figures is a superimposition of two models, based on either DS1 or DS2 [8]. The best correlating BLAST+ method (see above) was used throughout. Both the standard GLM and its logarithmized variant showed a substantial amount of overdispersion, as the residual deviances (1205431 for the GLM and 855004 for the log-variant) were significantly greater than the degrees of freedom (153). (For this reason, we chose a quasi-binomial error family instead of the binomial one as an additional means for reducing overdispersion thus providing significantly reduced confidence intervals; see Figure 6). The log-model had a smaller AIC (856739 compared to 1207167) and thus performed slightly better according to the models’ diagnostics than the standard GLM, in accordance with Figure 6. The standard linear regression models (Additional file 6, picture 11A) yielded an adjusted *R*^2^ of 0.89 for DS2 but only 0.86 for DS1, indicating that the linear model fits the data less the more observations are provided. The model for the arcsine- transformed data (Additional file 6, picture 11B) yielded approximately the same outcome (an *R*^2^ of 0.89 for the small data set and 0.84 for the large one). Additional file 7 holds DDH predictions for a series of distance values between 0 and 1 for all discussed models.

In Table 2 the error ratios of selected GBDP methods and distinct models are shown. Generalized linear models with log transformation provided the lowest mean error ratio and had the lowest deviation over distinct GBDP settings. The error ratios in predicting DDH at the 70% threshold subject to correlations between intergenomic distances and DDH are shown in Additional file 6 (picture 12). All subplots reveal a similar kind of structure. Moreover, as expected we observed that the error ratios increase with an increase in the correlation (again note that here a correlation of -1 is optimal). The log-GLMs, however, resulted in a higher correspondence between error ratio and Kendall correlation than the other types of models.

**Table 2:** Error ratios of selected GBDP methods

	**Model types**			
**GBDP settings**	**GLM**_***log***_	**GLM**	**LM**_***AS***_	**LM**
BLAT (*d*_6_)	0.045	0.058	0.052	0.052
BLAT (*d*_8_)	0.045	0.090	0.097	0.084
BLAST+ (*d*_6_)	0.090	0.065	0.071	0.052
BLAST+ (*d*_8_)	0.090	0.213	0.187	0.316
BLAST+ (*d*_4_)	0.039	0.052	0.052	0.052

Error ratios under different models and the full empirical data set. The here presented GBDP settings are a well-performing selection (see Kendall-related settings for DS1 in Table 1). The models are: linear, LM; linear with arcsine transformation, LM_*as*_; generalized linear, GLM; GLM with log-transformation, GLM_*log*_.

## Discussion

### Bootstrapping and jackknifing GBDP

With only minor differences between bootstrapping and jackknifing, the use of different algorithms had an obvious impact on the CIs of the resulting distances. The full implementation of the “coverage” algorithm allowed its application in connection with distance formulae *d*_4_ to *d*_9_ (so far it was restricted to *d*_0_ to *d*_3_). Its CVs and, accordingly, its CIs and those of the resulting DDH estimates, were two orders of magnitude smaller than the ones resulting from the “greedy” and “greedy-with-trimming” algorithms (Figure 4 and Additional file 6, picture 9). The CIs of the DDH values estimated with the “coverage” algorithm showed a typical quality of proportion data ([37], p. 515), i.e., very high as well as very low distances between two genomes cause a reduced amount of variation between the resampled distances.

That the “greedy” and “greedy-with-trimming” algorithm yielded substantially higher CVs and CIs, as well as an increase of CVs and CIs with decreasing distance (and, thus, increasing DDH similarity) is most likely caused by the fact that here sets of HSPs, not genome positions are resampled. The observed *Methanococcus* genome pair confirms this, as its comparison yielded few very long HSPs with a high impact on the resulting distance values during resampling (Additional file 6, picture 10). Indeed, we observed the overall tendency that more similar genomes not only result in more HSPs but also in HSPs with much less equally distributed lengths. Hence, for “greedy” and “greedy-with-trimming”, the resampling of HSPs probably leads to an overestimation of the uncertainty in distance estimation and thus cannot be recommended over the resampling in conjunction with the “coverage” algorithm, which by construction is not depending on HSPs during the resampling phase. The relative sizes of the CIs also implies that those from “coverage” bootstrapping or jackknifing are by an order of magnitude smaller than the CIs from the DDH prediction via models, and that those from “greedy” and “greedy-with-trimming” resampling are by an order of magnitude higher than the model-based CIs.

For practical purposes this indicates that in conjunction with “coverage”, bootstrapping and jackknifing GBDP can safely be omitted because the uncertainty it indicates is always substantially lower than the one indicated by the CIs of the model. This result is as expected, because genome sequencing results in a large number of characters – actually, the largest possible number of characters that can be sampled from an organisms –, and, if appropriately calculated, the uncertainty of intergenomic distances should be much lower than the one of wet-lab DDH experiments. Experimental errors in determining DDH in the wet lab, however, are likely to be responsible for the uncertainties in fitting the models for predicting DDH in silico.

### Models for DDH prediction

All previous studies [6-8] were based on simple linear regressions models (LMs) and did not consider data transformations. We observed that error ratios in DDH prediction, provided by LMs, are generally higher than those of the generalized linear models (GLMs). In addition to mere statistical parameters (such as overfitting, overdispersion, the AIC or *R*^2^), the size of the error ratio in predicting whether DDH values are above or below 70% is of particular interest. As expected, it was revealed that the smaller the correlation between DDH and intergenomic distance, the higher the error ratio (see Additional file 6, picture 12).

Moreover, the GLMs combined with the log-trans- formed explanatory variable yielded a higher consis- tency between the correlation coefficients and the prediction success at the 70% boundary, and the better correlating GBDP methods had, on average, a lower error ratio if combined with these log-GLMs instead of any of the other models. Additionally, GLMs, if applied as shown, guarantee that even DDH predictions based on extreme distance (or similarity) values are between 0% and 100%. For obvious reasons, models for species delimitation should be as exact as possible and, thus, LMs here at least be considered as problematic. The overdispersion detected when diagnosing GLMs was presumably due to distinct pairs of strains sharing identical intergenomic distance values but at the same time showing distinct DDH values. This effect is called “unmodeled heterogeneity” and could also result from clustering of the DDH measurements ([48], p. 52–61), as observed in our data set. A switch to a “complementary log-log”-link function, as suggested in ([37], p. 594), didn’t further improve the model (data not shown).

The enlarged data set provided a globally increased significance of the inferred results. The comparison of selected GBDP methods applied to either the old [8] or the new data set, however, revealed only minor differences in the parameters estimated by the statistical models we tested.

Both theoretical and empirical results thus favor GLMs over standard linear-regression models for obtaining in-silico DDH replacement methods. Its improved DDH prediction capabilities offer GBDP as a quick and now even more reliable alternative to the DDH wet-lab technique, thus moving further forward within the transition process to a genome-based taxonomic gold standard.

### The recommended GBDP method

In principle, multiple optimality criteria could be applied for selecting a GBDP variant that works best in DDH prediction, depending on the users’ priorities. The newly completed “coverage” algorithm, however, can unanimously be recommended, because it yielded the best correlations for both formula *d*_6 _(in general, and particularly combined with BLAST+ using a word length of 46 and no e-value filtering) and formula *d*_4 _(in general, and particularly combined with BLAST+ using a word length of 38 and no e-value filtering). When dealing with incomplete genomes it is highly recommended to use formula *d*_4_, as it is independent of sequence length, and thus not directly affected by the removal of HSPs due to the removal of parts of the genome [8] (see also Additional file 3). Here, formula *d*_4 _resulted in worse Kendall correlations but better Pearson correlations and error ratios at the 70% boundary than *d*_6_, as observed earlier [8].

Regarding local-alignment programs, only BLAT performed better than BLAST+ combined with optimized settings, and only slightly so. BLAST+ ’s optimal initial word length setting of 38 or 46 allows for comparatively quick genome-genome comparisons, because the intergenomic search space is significantly reduced compared to the default value of 11, resulting in a lower execution time. A higher initial word length results in a lowered sensitivity of the local-alignment program, which had a positive effect on the correlation outcome, as previously reported [8]. This is in agreement with the fact that BLAT, which is a considerably less sensitive alternative to BLAST, overall performed best [8]. All in all, we conclude that the default setting for the novel GBDP implementation should be BLAST+ combined with *d*_4 _and the accordingly optimized settings regarding word length and e-value filtering, and that the corresponding log-GLM model should be used for predicting DDH including CIs. Moreover, the situations in which a user might instead favor BLAT over BLAST+ and/or *d*_6_ over *d*_4_ are straightforward to identify. All these recommendations can now be directly utilized via our updated web service (GGDC 2.0) at http://ggdc.dsmz.de.

### Beyond pairwise distances

Since the dawn of computer-based approaches to phylogenetics, researchers were trying to devise solutions for assessing statistical support of the inferred phylogenies [49-52]. Apparently, branches lacking sufficient support should not be overestimated regarding the explanation and visualization of evolutionary events. Particularly bootstrapping and jackknifing [53] are widely-used solutions for this kind of question and can be applied to both aligned molecular sequences (multiple sequence alignments) and matrices of phenotypic characters. Here, resampling is applied to the characters, usually present as columns of a matrix whose rows represent the organisms, phylogenetic inference is applied to the resampled matrix, and finally a majority-rule consensus tree is calculated from the trees from all replicates [51]. If distance methods for phylogenetic inference such as neighbour-joining [53] are used in such a scenario, within each replicate a distance matrix is computed from a character matrix that has been resampled at once.

In contrast, distance methods that avoid the construc- tion of a character matrix would need to apply boots- trapping or jackknifing to each pairwise comparison independently. For instance, [54] developed a method that relies on pairwise sequence alignment only; here, maximum-likelihood distances are inferred, and bootstrapped independently, from the alignments of all pairs of sequences. To highlight the conceptual difference, the procedure was called “pseudo-bootstrapping”, and it was demonstrated to be conservative compared to bootstrap analysis of multiple sequence alignments [54].

Apparently, GBDP’s new bootstrapping and jackknifing facilities would also yield such a pseudo-bootstrapping approach if it was applied to phylogenetic problems. In particular, sequence comparison is conducted independently for all genome pairs involved, and the sets of HSPs or the coverage vectors – on which each pairwise comparison is based – never form a common character matrix [12,13]. Bootstrapping and/or jackknifing would just add the individual resampling of these independently constructed sets of HSPs or coverage vectors. An advantage to phylogenomics provided by GBDP-bootstrapping over supermatrix approaches (which concatenate alignments of individual orthologous genes; see [4] for an overview of phylogenomic methods in the context of microbial taxonomy) is that the calculation of bootstrapped or jackknifed distances could be done incrementally, and only the phylogenetic inference from all formed distance matrices would need to be done after each update of the set of organisms of interest.

For this reason, GBDP with resampling could be a faster and resource-saving alternative to more compute-intense phylogenomics methods, particularly because GBDP can as well be applied to sequences from proteomes [13]. Besides, it easily copes with various phylogenetic problems such as paralogous genes [12], low-complexity regions [13] and unbalanced genome/proteome sizes [8]. However, whereas this study already presented evidence that “coverage” should be preferred over “greedy” and “greedy-with-trimming” if coupled with bootstrapping or jackknifing, it is a partially open question whether, and under which conditions, resampling proteome-based GBDP[13] should be preferred over analyzing nucleotide sequences this way [12]. Even though it is likely that the deeper branches of the phylogeny can only be resolved based on amino acid sequences GBDP[13], in-depth comparisons of the performance of GBDP- bootstrapping/jackknifing with more common phylogenomics methods, as well as similar methods that are also based on resampling HSPs [55], are still needed.

Nevertheless, that a single method can be applied to both genome-based species delimitation and phylogenomic inferences at other taxonomic levels, and that it can be coupled with the assessment of statistical significance at one level, already strongly indicates that GBDP is an important tool in the transition process to genome-based gold standards at all taxonomic levels. A tighter coupling between phylogenetic inference and the assignment of taxonomic ranks might also help to overcome what we regard as the most severe theoretical limitation of the DDH 70% rule: that a taxon defined as all organisms whose similarity to a type organism is above a certain threshold is never guaranteed to form a monophyletic group [53]. The practical limitations of the wet lab-based DDH, however, already seem to have been overcome.

## Conclusions

This update on the GBDP method is an important enhancement, not only because existing features of the software have been improved but particularly because novel features have been added. Whereas GBDP was already shown to yield better correlation results in DDH prediction than ANI [6] in an earlier correlation study [8], we can also confirm this with respect to the JSpecies[7] implementation. Since taxonomists generally consider these approaches as potential “next generation” replacements for the traditional and currently still dominating wet-lab method [3,4], up to now these approaches could not be used to determine the CI of intergenomic distance measures, thus rendering the latest installment of the GBDP method to be the first one supporting that feature. This is crucial because numeric estimations from empirical data (such as wet-lab DDH values) always yield a certain degree of uncertainty, and it is thus commonplace in statistics to provide measures of variation and confidence.

By introducing (i) bootstrapping and jackknifing to the GBDP approach, (ii) better performing DDH prediction models and the CIs they provide, and, (iii) direct calculation of the probability that an intergenomic distance yielded a DDH larger than 70%, the here presented methods provide an attractive alternative to the wet-lab DDH for current taxonomic techniques. The addition of novel distance functions (by completing the implementation of the “coverage” distances) was also beneficial here, particularly in conjunction with the novel resampling techniques and with respect to the resulting correlations with DDH.

## Competing interests

The authors declare that they have no competing interests.

## Authors’ contributions

JMK participated in the design of the study, carried out the experiments, performed the (statistical) analysis and wrote the manuscript. AFA contributed software methods to this study and helped carrying out the experiments. MG and HPK designed and conceived the study. MG also participated in writing the manuscript. All authors read and approved the final manuscript.

## Supplementary Material

Additional file 1 **Empirical data sets.** CSV file holding the empirical data sets used in this study. The file can be accessed with spreadsheet programs (e.g., Excel, OpenOffice or LibreOffice) or any given text editor. Click here for file

Additional file 2 **Overview on the examined GBDP input parameters.** PDF file holding a table about all GBDP settings/input parameters tested in this study. Click here for file

Additional file 3 **All distance formulae used by **GBDP**.** PDF file holding a table with all distance formulas implemented in GBDP. Click here for file

Additional file 4 **Results of the correlation analysis.** Spreadsheet in Open Document Format (ODS) that can be accessed via common spreadsheet programs (e.g., Excel, OpenOffice or LibreOffice). The Spreadsheet contains several tabs, each one holding the results for the data sets DS1-DS4 (see Materials and Methods). Click here for file

Additional file 5 **Input for the multiple linear regression analysis.** Spreadsheet in Open Document Format (ODS) that can be accessed via common spreadsheet programs (e.g., Excel, OpenOffice or LibreOffice). The Spreadsheet contains the input data required for reproducing the multiple linear regression analysis. Click here for file

Additional file 6 **Additional figures.** PDF file holding all figures that did not fit in the main manuscript, although these help to further elucidate the study and its results. Click here for file

Additional file 7 **DDH prediction-based intergenomic distances.** CSV file holding sample DDH predictions under different statistical models as analyzed in this study. The file can be accessed with spreadsheet programs (e.g., Excel, OpenOffice or LibreOffice) or any given text editor. Click here for file
