# Supplementary material for: Genome sequence-based species delimitation with confidence intervals and improved distance functions
Source: BMC Bioinformatics. 2013 Feb 21;14:60. doi: 10.1186/1471-2105-14-60 (PMC3665452; doi:10.1186/1471-2105-14-60)
Supplement: Additional file 2 — Overview on the examined GBDP input parameters. PDF file holding a table about all GBDP settings/input parameters tested in this study. [file 1471-2105-14-60-S2.pdf]

Supplementary Table 2. Overview on the examined GBDP input parameters

Local alignment program	Command-line options	E-value threshold	Mnemonic
BLAT (BT)	minScore=0 minIdentity=0 tileSize=8	10 (“NF”), 10^{-2} (“F-2”)	BT_ms0.id0.TS8_NF and BT_ms0.id0.TS8_F-2
	minScore=30 minIdentity=50 tileSize=11 (default)	10, 10^{-2}	BT_ms30.id50_NF and BT_ms30.id50_F-2
	minScore=30 minIdentity=90 tileSize=12	10, 10^{-2}	BT_ms30.id90.TS12_NF, BT_ms30.id90.TS12_F-2
BLAST+ (BP)	WordLength=7, 9, 11-20, 22, 24, 26, 28, 30, 32, 34, 36, 38, 40, 42, 44-46, 48, 50	10, 10^{-1}, 10^{-2}, 10^{-8}	BP_WLx_F-y (x=word length, y=exponent of e-value filter)
NCBI-BLAST (NB)	default settings	10, 10^{-2}	NB_NF / F-2
WU-BLAST (WU)	hspsepSmax=50 hspsepQmax=50	10, 10^{-2}	WU_S50.Q50_NF and WU_S50.Q50_F-2
BLASTZ (BZ)	K=2000 (2500, 3000, 3500)	10, 10^{-2}	BZ_K2000_NF / F-2 BZ_K2500_NF / F-2 BZ_K3000_NF / F-2 BZ_K3500_NF / F-2
MUMmer (MU)	-mum -minMatchLength=x (with x=20, 30, 40, 44 or 50)	-	MU_mum20, MU_mum30, MU_mum40, MU_mum44, MU_mum50
	-mumreference -minMatchLength=x (with x=20, 30, 40, 44 or 50)	-	MU_ref20, MU_ref30, MU_ref40, MU_ref44, MU_ref50
	-maxmatch -minMatchLength=x (with x=20, 30, 40, 44 or 50)	-	MU_max20, MU_max30, MU_max40, MU_max44, MU_max50

Overview of local-alignment programs and their settings assessed in the current study. The most parameter settings were tested for BLAST+ since it is one of the most advanced local alignment tools available [26]. MUMmer does not support e-value filtering since it is restricted to exact matches (MUMs). In total 4350 distinct GBDP variants were tested (compare Supplementary Table S4) thus resulting in about 136 million individual genome comparisons which had to be conducted (i.e., 4350 settings · 156 genome pairs · (1 original distance + 100 bootstrap replicates + 100 jackknife replicates)).
