# Supplementary material for: Genome sequence-based species delimitation with confidence intervals and improved distance functions
Source: BMC Bioinformatics. 2013 Feb 21;14:60. doi: 10.1186/1471-2105-14-60 (PMC3665452; doi:10.1186/1471-2105-14-60)
Supplement: Additional file 3 — All distance formulae used by GBDP. PDF file holding a table with all distance formulas implemented in GBDP. [file 1471-2105-14-60-S3.pdf]

Supplementary Table 3. All distance formulae used by GBDP

Group	ID	Formula	Motivation
A	d_0	$1 - \frac{H_{XY} + H_{YX}}{\lambda(X,Y)}$	Proportion of genomes covered by HSPs [12].
A	d_1	$1 - \frac{H_{XY} + H_{YX}}{\lambda_{\min}(X,Y)}$	Like d_0 but robust against huge differences in genome size [12].
A	d_2	$-\log\left(\frac{H_{XY} + H_{YX}}{\lambda(X,Y)}\right)$	Variant of d_0 rescaled for phylogenetic inference [12].
A	d_3	$-\log\left(\frac{H_{XY} + H_{YX}}{\lambda_{\min}(X,Y)}\right)$	Variant of d_0 rescaled for phylogenetic inference [12].
B	d_4	$1 - \frac{2 \cdot I_{XY}}{H_{XY} + H_{YX}}$	Total number of identical base pairs within HSPs relative to total coverage by HSPs [12].
B	d_5	$-\log\left(\frac{2 \cdot I_{XY}}{H_{XY} + H_{YX}}\right)$	Variant of d_4 rescaled for phylogenetic inference [12].
C	d_6	$1 - \frac{2 \cdot I_{XY}}{\lambda(X,Y)}$	Total number of identical base pairs within HSPs relative to genome size [14].
C	d_7	$1 - \frac{2 \cdot I_{XY}}{\lambda_{\min}(X,Y)}$	Like d_6 but robust against huge differences in genome size [8].
C	d_8	$-\log\left(\frac{2 \cdot I_{XY}}{\lambda(X,Y)}\right)$	Variant of d_6 rescaled for phylogenetic inference [14].
C	d_9	$-\log\left(\frac{2 \cdot I_{XY}}{\lambda_{\min}(X,Y)}\right)$	Variant of d_7 rescaled for phylogenetic inference [8].

Distance formulae can be subdivided into three groups A, B and C based on the type of denominator. Each formula addresses specific intergenomic relationships. All definitions used within the formulae are explained in Materials and Methods. Formulae of type C preserve the most information, among these, d_9 performed best in a phylogenetic context. But only formulae of type B are robust against the use of incompletely sequenced genomes [8]. When using the web service on `http://ggdc.gbdp.org` only formulae d_0, d_4 and d_6 (named formulae 1, 2 and 3 in the result e-mail) are reported since these are most relevant in the context of digital DDH [16].
