# Supplementary material for: Genome sequence-based species delimitation with confidence intervals and improved distance functions
Source: BMC Bioinformatics. 2013 Feb 21;14:60. doi: 10.1186/1471-2105-14-60 (PMC3665452; doi:10.1186/1471-2105-14-60)
Supplement: Additional file 6 — Additional figures. PDF file holding all figures that did not fit in the main manuscript, although these help to further elucidate the study and its results. [file 1471-2105-14-60-S6.pdf]

S6 - Supporting material for "Genome sequence-based species delimitation with confidence intervals and improved distance functions" by Jan P. Meier-Kolthoff, Alexander F. Auch, Hans-Peter Klenk and Markus Göker

File: S6_additional_figures.pdf

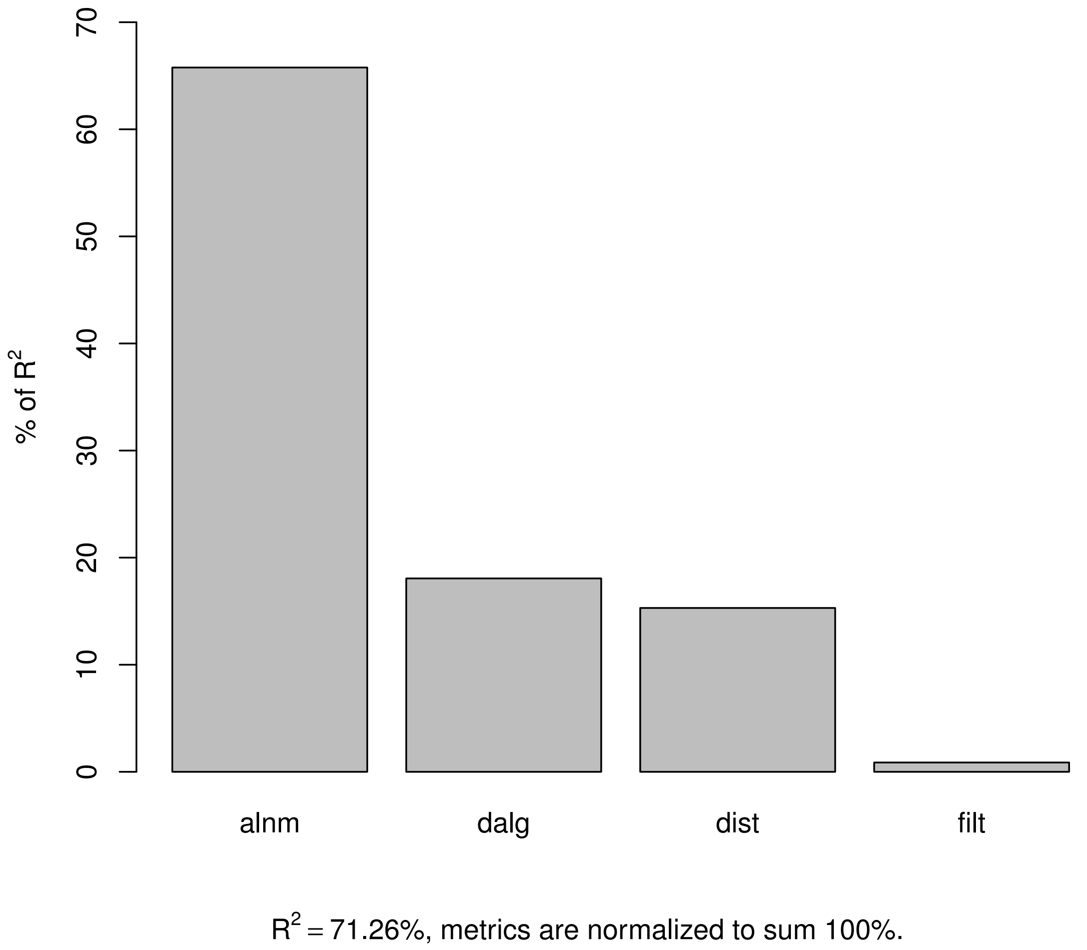

Supplementary Picture 1. Relative-importance indexes for a multiple linear regression model with Kendall correlation values as response. Each predictor variable's relative importance index [40] was computed on the basis of a standard, non-interacting linear model. The higher a variable's contribution to the coefficient of determination, the higher its relative importance is. It turns out that the predictor variable "e-value filter method" (filt) has an insignificant contribution to the overall coefficient of determination (R^2). Predictor variables "distance algorithm" (dalg) and "distance formula" (dist) have both a contribution of around 15%, whereas the one for "alignment method" (alnm) is around 65%. The "lmg" metric was used for this analysis.

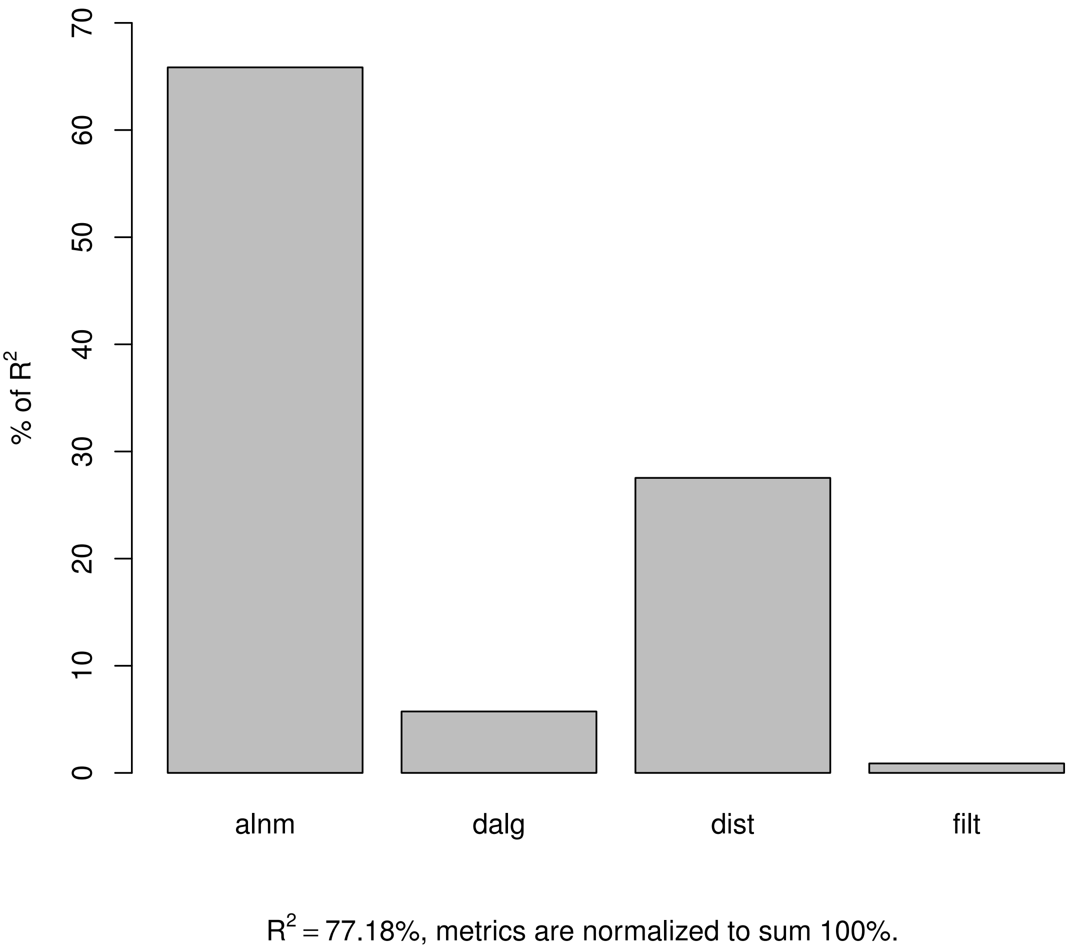

Supplementary Picture 2. Relative importance indexes for a multiple linear regression model with Pearson correlation values as response variable. For details on this kind of analysis, see last figure caption. It turns out that predictor variable "e-value filter method" (filt) has an insignificant contribution to the overall coefficient of determination (R^2). Here, the contribution of the algorithms "coverage", "trimming" and "greedy" (predictor variable named "dalg") to R^2 is small compared to the results with Kendall's correlation coefficients. The predictor "distance formulae" (dist) contribute to R^2 by around 30% which is approximately twice the amount observed in the results with Kendall's correlation coefficients.

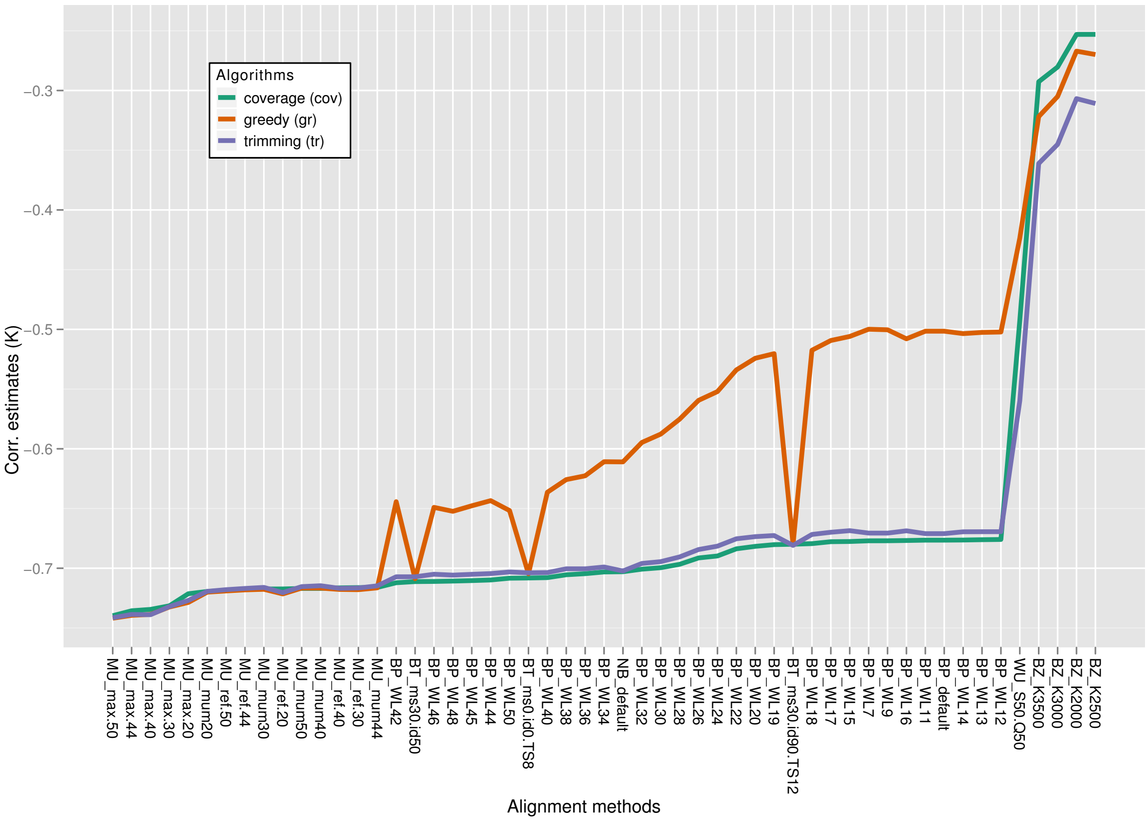

Supplementary Picture 3. Interaction effects between predictor variables "alignment methods" and "algorithm" with Kendall correlations as response variable. Kendall correlations were calculated between DDHs and intergenomic distances for the data set DS1. In the picture, alignment methods are sorted ascending according to the correlation estimates of the "coverage" predictor. Whereas algorithms "coverage" and "trimming" had an almost equal and negative effect (i.e., made the correlation coefficient more negative) on the predictor "alignment methods", "greedy" had a less pronounced effect for most methods. A further look at the models' coefficients revealed that `GBDP` methods incorporating `BLAST+` with initial word lengths of 38-50 had an average impact of about -0.5 on the correlation coefficient. The `MUMmer`- and `BLAT`-related variants performed even better in this respect, achieving approx. -0.6.

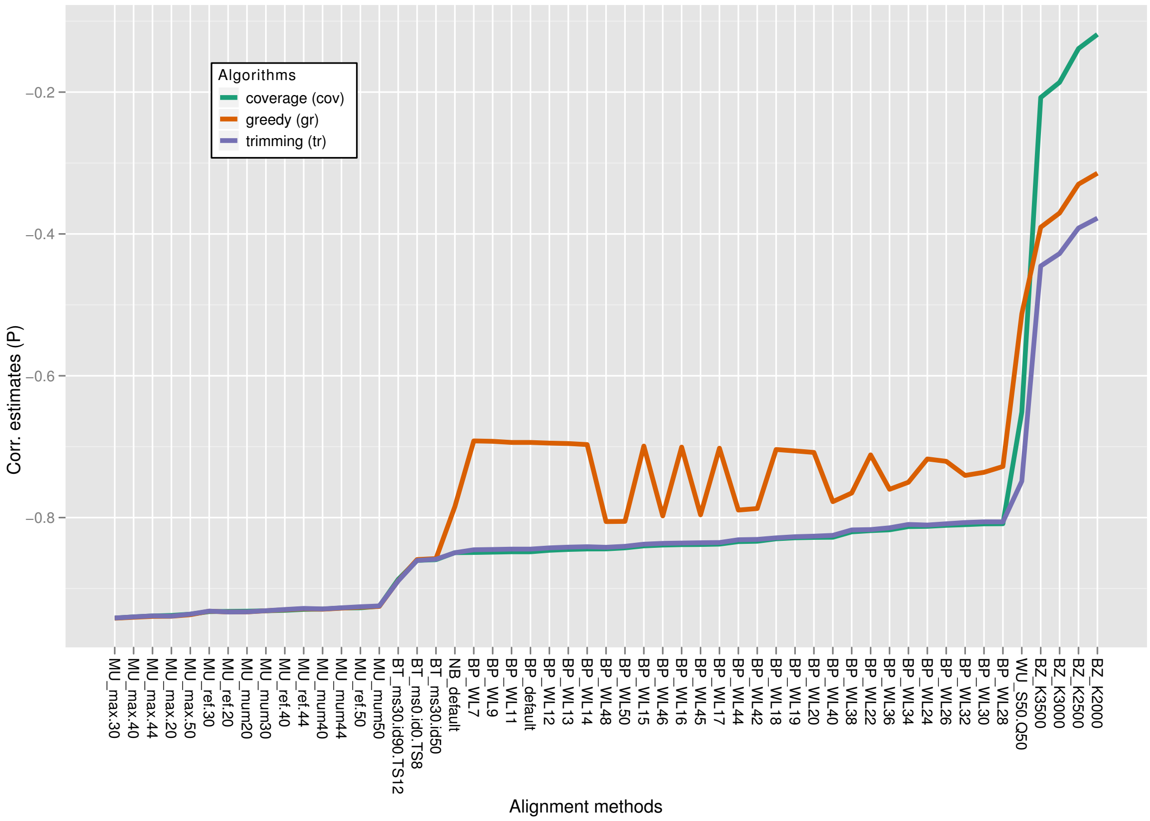

Supplementary Picture 4. Interaction effects between predictor variables "alignment methods" and "algorithm" with Pearson correlations as response variable. Pearson correlations were calculated between DDHs and intergenomic distances for the data set DS1. In the picture, alignment methods are sorted ascending according to the correlation estimates of the "coverage" predictor. Whereas algorithms "coverage" and "trimming" had an almost equal and substantial negative effect (i.e., made the correlation coefficient more negative) on the predictor "alignment methods", "greedy" had a less pronounced effect for most methods. A further look at the models' coefficients revealed that `GBDP` methods incorporating `BLAST+` with initial word lengths of 38-50 had an average impact of about and -0.7 on the correlation coefficient. The `MUMmer`- and `BLAT`-related variants performed even better in this respect, achieving approx. -0.9.

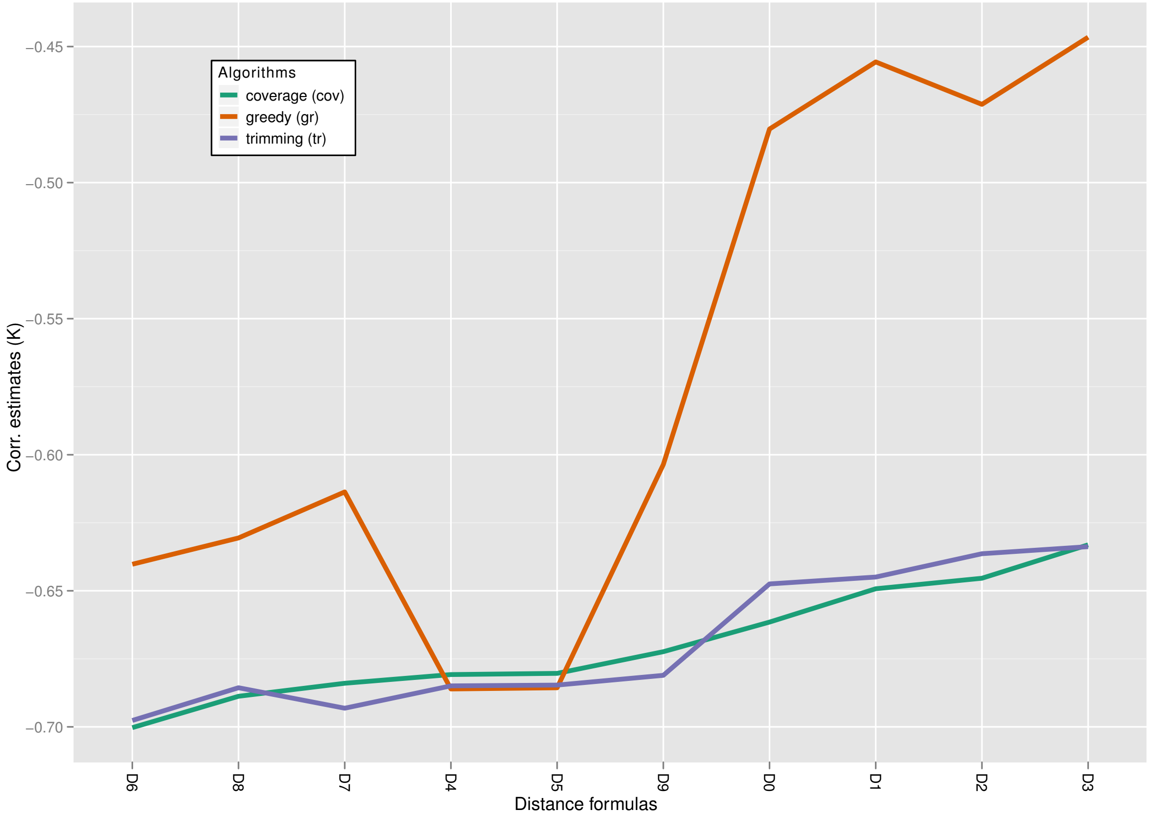

Supplementary Picture 5. Interaction effects between predictor variables "distance formula" and "algorithm" with Kendall correlations as response variable. Kendall correlations were calculated between DDHs and intergenomic distances for the data set DS1. Distance formulae are sorted ascending according to the correlation estimates of the "coverage" predictor. Whereas algorithms "coverage" and "trimming" had an almost equal and substantial negative effect (i.e., made the correlation coefficient more negative) on the predictor "distance formulae", "greedy" had a less pronounced effect for most formulae.

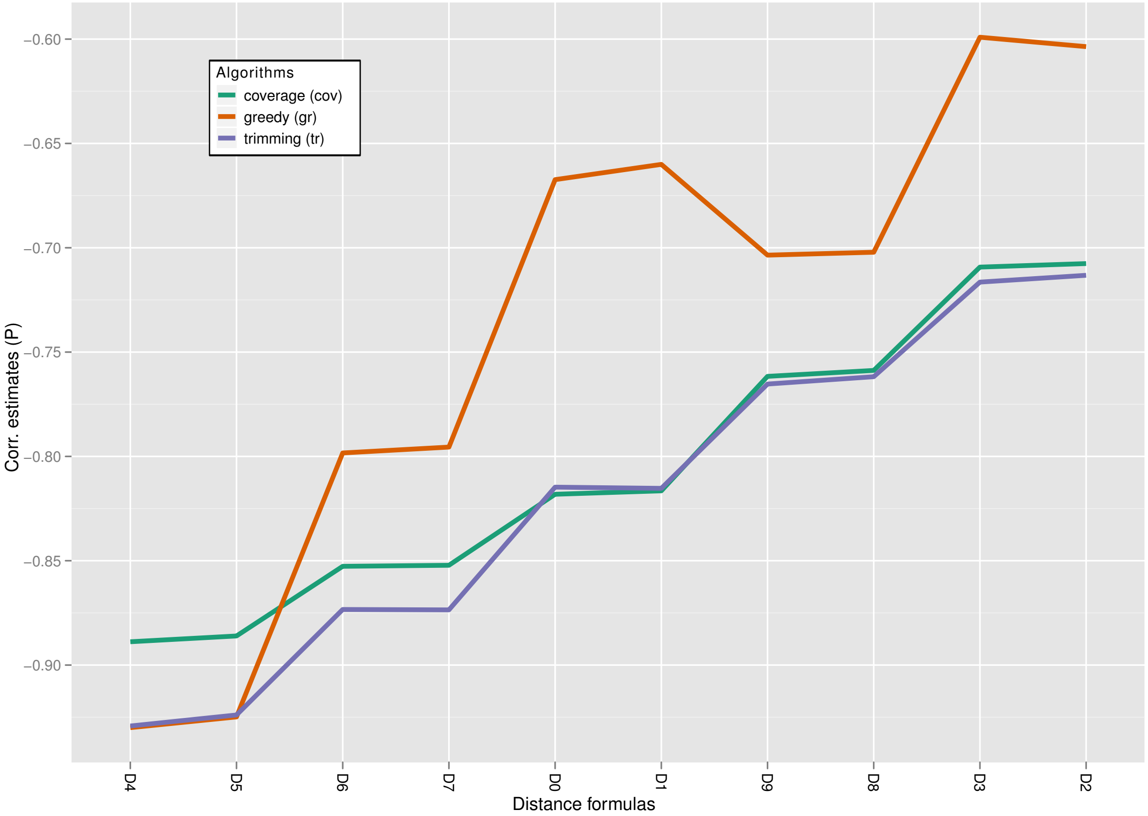

Supplementary Picture 6. Interaction effects between predictor variables "distance formula" and "algorithm" with Pearson correlations as response variable. Pearson correlations were calculated between DDHs and intergenomic distances for data set DS1. In the picture, distance formulae are sorted ascending according to the correlation estimates of the "coverage" predictor. Whereas algorithms "coverage" and "trimming" had an almost equal and substantial negative effect (i.e., made the correlation coefficient more negative) on the predictor "distance formulae", "greedy" had a less pronounced effect for most formulae (except D4 and D5).

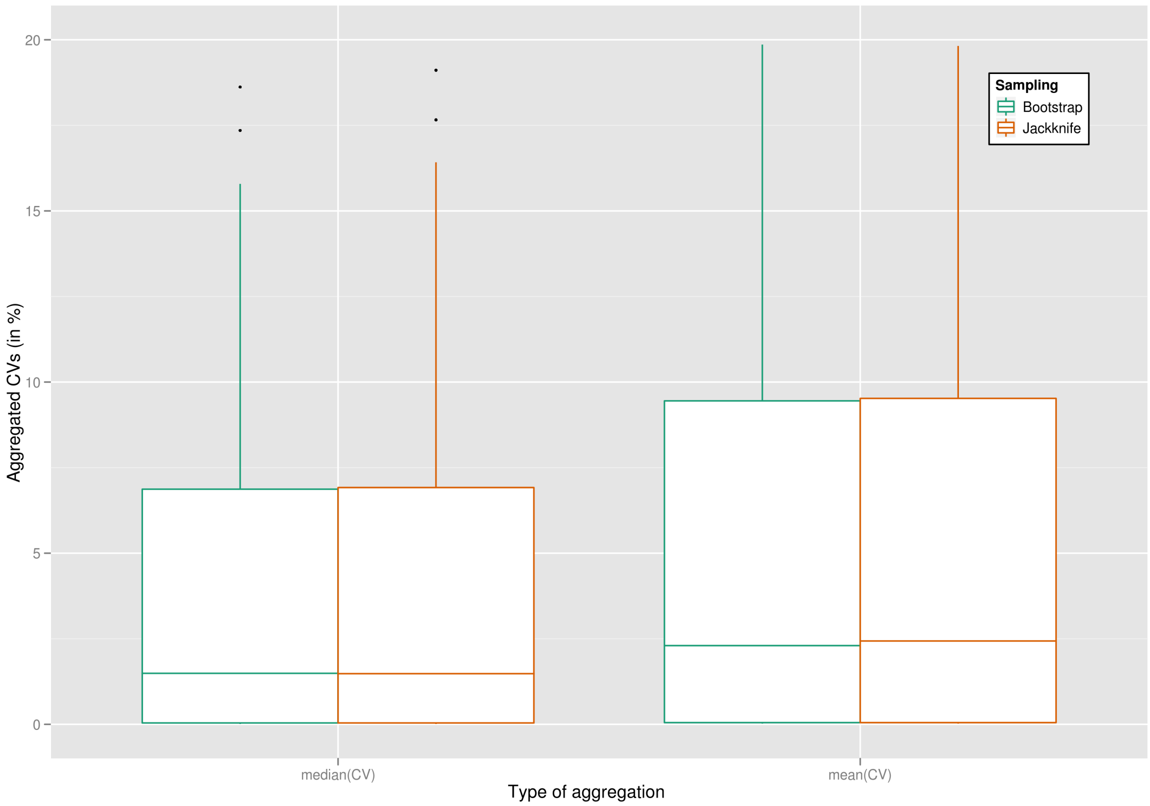

Supplementary Picture 7. Overall distributions of the coefficients of variation as computed for all 4350 distinct `GBDP` settings tested and their respective distance replicates. The medians of both distributions revealed little differences between bootstrapping and jackknifing. Rather, the way the coefficients of variation were aggregated for each `GBDP` setting (either median or mean) slightly affected the overall median of the resulting distributions.

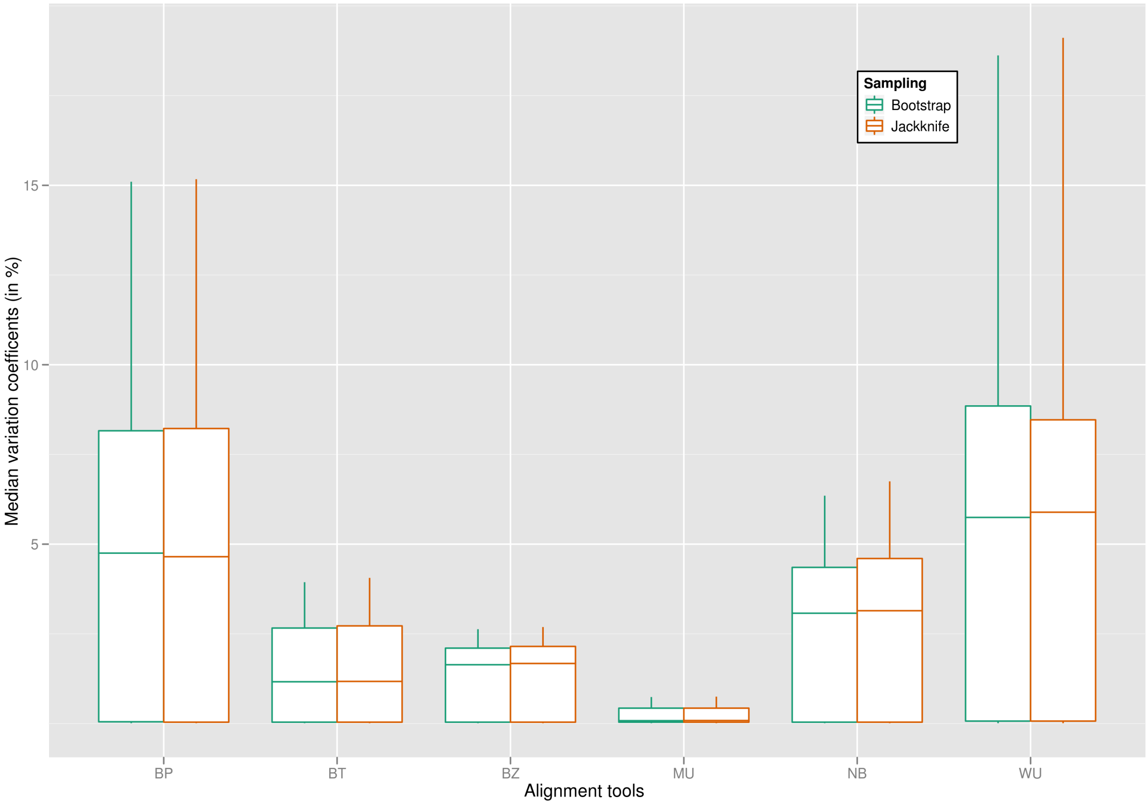

Supplementary Picture 8. Distributions of the coefficients of variation grouped by local-alignment method. The coefficients of variation (CV) of all 4350 distinct `GBDP` settings (and their respective distance replicates) were aggregated by calculating the median. An average, `MUMmer` (MU) yielded the lowest median coefficient of variation of all local-alignment methods tested. The two boxplots for the `BLAST+` method (BT) are much broader, apparently due to the wide range of diverse `BLAST+` settings tested in this study.

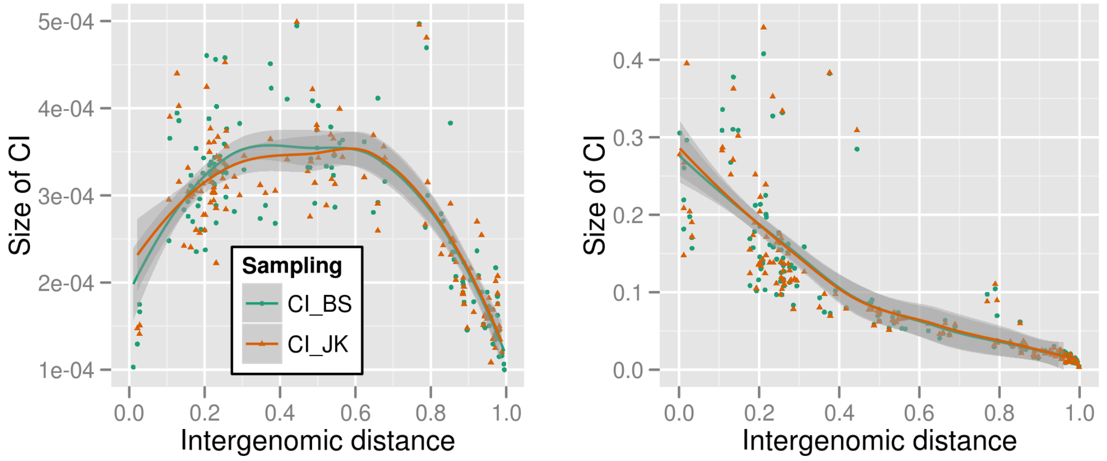

Supplementary Picture 9. Results of the analysis of distance confidence intervals (CIs) for a selected, well performing GBDP method using the coverage and trimming algorithms. The subfigures show the relationship between intergenomic distance point estimates and the sizes of their CIs obtained under either the coverage (left) or trimming algorithm (right). **Left:** Under both bootstrapping (BS) and jackknifing (JK, with a deletion ratio of 50%) the width of the CIs decreased towards the lower and upper bounds, as expected for proportion data. **Right:** The "trimming" algorithm yields, on average, much broader CIs (note that the two subfigures are not drawn to scale). Their sizes decrease with increasing distance point estimate. The broadest confidence interval observed (≥ 0.4) occurred at a point estimate of around 0.2 and was associated with the genome pair *Methanococcus maripaludis* C6 and *Methanococcus maripaludis* C7. The two genomes shared 162 HSPs whose lengths were unevenly distributed: 61 had a length below 1kb, whereas 42 were significantly above 10kb in length. This genome pair was part of a larger analysis that addressed the relationship between the number of HSPs and average and median HSP lengths to the corresponding intergenomic distance estimate (see next picture).

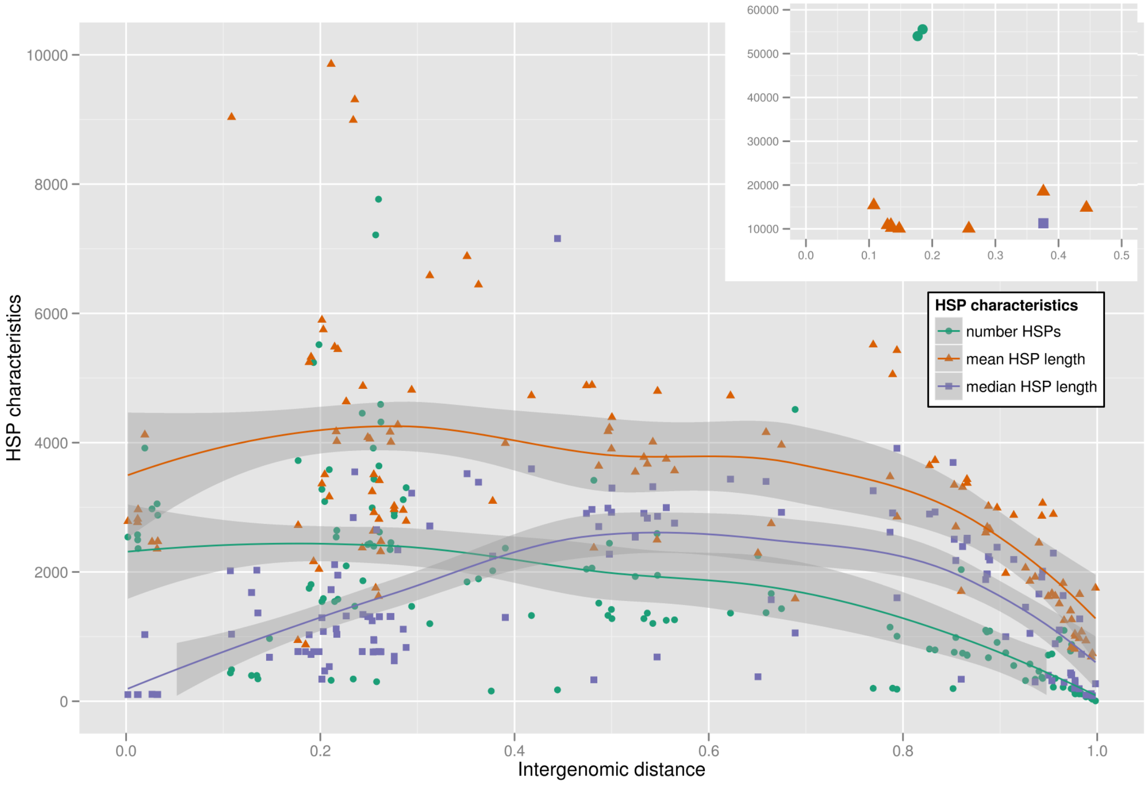

Supplementary Picture 10. Relationship between intergenomic distance and the numbers as well as lengths of underlying HSPs. This type of analysis was conducted to provide insight into genome characteristics potentially affecting `GBDP`'s bootstrapping and jackknifing behavior. The HSP sets from all 155 genome pairs (data set DS1) were analyzed in detail for a well-performing `GBDP` method (see Table 1). Three characteristics were assessed for each HSP set: the total number of HSPs, the average HSP length and the median of the HSP lengths. HSP lengths between closely related genomes (small intergenomic distance) are rather unevenly distributed. Bootstrap and jackknife replicates of the same underlying set of HSPs would thus rather strongly differ regarding the lengths of the selected HSPs, most likely yielding broad distance confidence intervals (see last figure). The small subplot shows some outliers (same scale) that have been intentionally omitted in the main plot. Green dots in that subplot refer to genome pairs from *Methanococcus maripaludis* strains C5, C6 and C7.

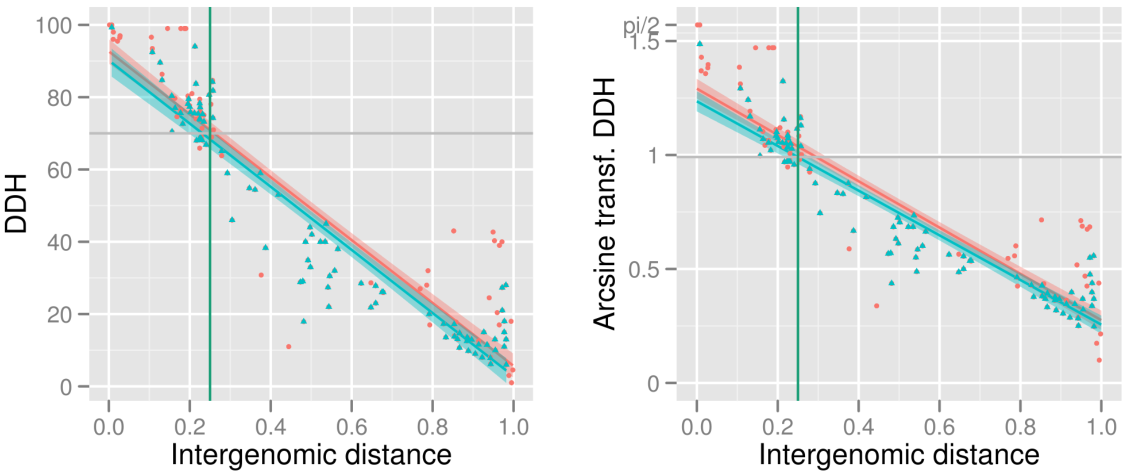

Supplementary Picture 11. Comparison of linear models and data transformations for DDH prediction. All model fits were based on intergenomic distances calculated by the same, well-performing `GBDP` method: `BLAST+` (word length=46), no e-value filtering, "coverage" algorithm and distance formula d_6. The model was either inferred from (i) the complete data set DS1 (red curve) or (ii) the reduced data set [8] DS2 (blue curve). Thus, the distance-DDH pairs marked by blue triangles occurred only in DS2, those marked by red circles also in DS1. The green vertical line indicates the 50% probability threshold as calculated by the GLM for binary response data (see Figure 5). **A:** Linear models using DDH as response and untransformed distances as predictor variable. **B:** Linear models using arcsine-transformed DDH values as response and untransformed distances as predictor variable.

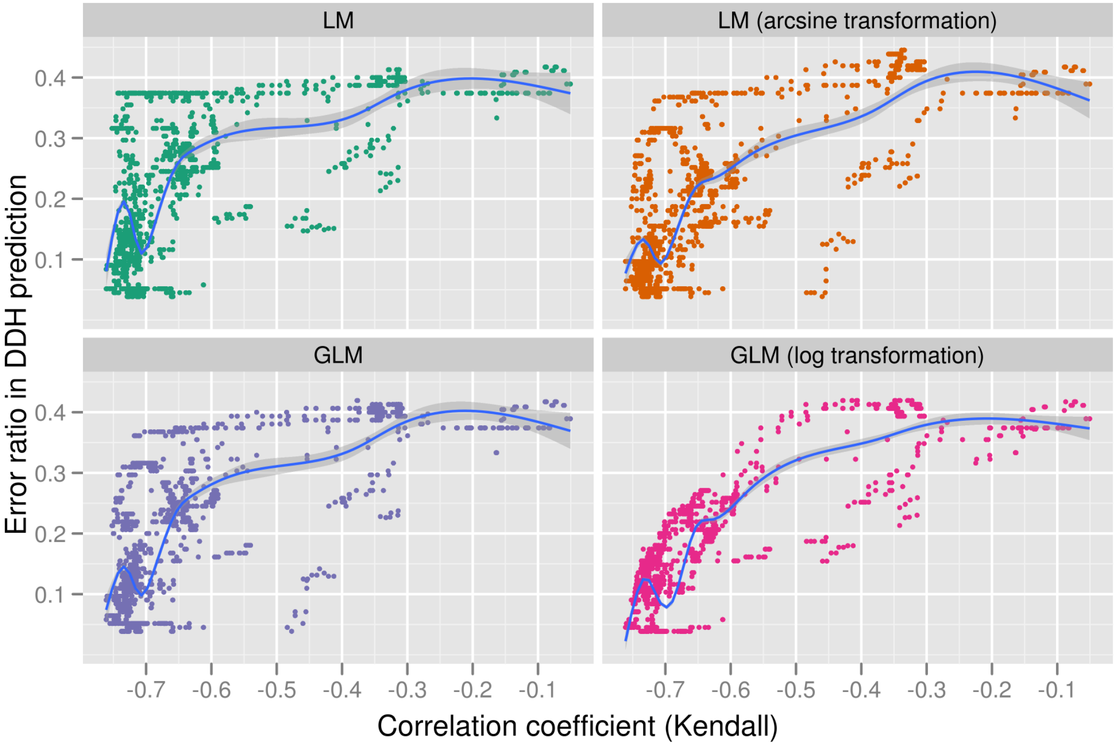

Supplementary Picture 12. Error ratios of DDH prediction in dependency of the `GBDP` correlations and the four model types. Error-ratios of predictions at the 70% boundary were calculated based on four types of models, linear models (LM) with or without arcsine transformation of the response variable and generalized linear models (GLMs) with or without log transformation of the predictor variable. Error ratios were determined for all tested `GBDP` methods and plotted over their Kendall correlation coefficients. Loess smoothers (blue curves) with their respective confidence intervals were added to each subplot. Apparently the error ratios increase with increasing (i.e., worse) correlations for all four models, but the relationship is much clearer for the log-GLMs than for the other models. For instance, the confidence bands of the Loess smoother are clearly smaller compared to the other models. Some selected error-ratios are found in Table 2.
